# Broad Bean (*Vicia faba* L.) Induces Intestinal Inflammation in Grass Carp (*Ctenopharyngodon idellus* C. et V) by Increasing Relative Abundances of Intestinal Gram-Negative and Flagellated Bacteria

**DOI:** 10.3389/fmicb.2018.01913

**Published:** 2018-08-17

**Authors:** Zhifei Li, Ermeng Yu, Guangjun Wang, Deguang Yu, Kai Zhang, Wangbao Gong, Jun Xie

**Affiliations:** ^1^Key Laboratory of Tropical and Subtropical Fishery Resource Application and Cultivation, Pearl River Fisheries Research Institute, Chinese Academy of Fishery Sciences, Guangzhou, China; ^2^Guangdong Ecological Remediation of Aquaculture Pollution Research Center, Guangzhou, China

**Keywords:** gut inflammation, flagellin, gram-negative bacteria, grass carp, broad bean

## Abstract

Constant consumption of broad bean (*Vicia faba* L.) induces intestinal inflammation and reduces growth rate in grass carp (*Ctenopharyngodon idellus* C. et V). However, the mechanisms underlying these effects are unclear. In mammalian models of inflammatory bowel disease (IBD), endotoxin and flagellin cause intestinal inflammation through upregulation of tumor necrosis factor (TNF)-α expression. We therefore speculated that broad bean consumption alters intestinal microbiota composition, thereby increasing the relative abundance of endotoxin-producing Gram-negative and flagellated bacteria and resulting in upregulation of TNF-α and intestinal inflammation in grass carp. We tested this hypothesis by comparing intestinal microbiota compositions of grass carp fed broad bean (GCBB), hybrid giant napier (*Pennisetum sinese* Roxb, GCHG), or formula feed (GCFF) by 16S rRNA gene sequencing. We also performed a histological analysis of the intestinal inner wall by scanning electron microscopy and measured intestinal wall and serum concentrations of TNF-α. Our results revealed epithelial cell damage including microvillus effacement and synechia along with increased TNF-α levels in the intestinal wall in the GCBB group as compared to the GCHG and GCFF groups. The relative abundances of Gram-negative and flagellated bacteria were also higher in the GCBB group than in the GCHG and GCFF groups; this was accompanied by upregulation of genes expressing endotoxin and flagellin in intestinal microbiota. Thus, broad bean-induced intestinal inflammation in grass carp shares features with IBD. Our findings demonstrate that the microbiome in fish is directly influenced by diet and provide a reference for deconstructing host–intestinal microbiota interactions.

## Introduction

Grass carp (*Ctenopharyngodon idellus* C. et V) fed broad bean (*Vicia faba* L., grass carp fed broad bean, GCBB) has become a popular food in China owing to its more pleasing texture compared to grass carp fed a conventional diet. In 2012, the harvest volume of GCBB was over 22,100 tons in Guangdong Province, China (Zhang et al., 2015). GCBB exhibits increased muscle hardness and crispiness as compared to normal grass carp (Yu et al., 2017) due to a 36.7% higher collagen content in muscle (Liu et al., 2011). However, constant consumption of broad bean by the carp causes intestinal mucosal injury resulting from perpetual inflammation (Zhang, 2015), although the underlying mechanism is unclear.

Intestinal inflammation is often associated with alterations in intestinal microbiome composition (Hughes et al., 2017). These changes are not only a consequence but also a cause of diseases such as inflammatory bowel disease (IBD) in mammals (Chassaing et al., 2017). Bacteria-derived flagellin and endotoxin are two major proteins that induce host intestinal inflammation. The former, which is the main component of the bacterial flagellum, can activate a mucosal inflammatory response (Gewirtz et al., 2001a,b; Zeng et al., 2003; Huang et al., 2018), for instance by translocation of the protein across the intestinal epithelium in *Salmonella* (Gewirtz et al., 2001a; Zeng et al., 2003). Bacterial flagellin activates basolaterally expressed Toll-like receptor (TLR)-5 to induce epithelial pro-inflammatory gene expression (Gewirtz et al., 2001b). Endotoxin (i.e., lipopolysaccharide) is a major component of the outer membrane of Gram-negative bacteria (Zhang et al., 2012; Sipka and Bruckner, 2014), which show increased abundance in most intestinal inflammatory diseases in mammals (Mazmanian et al., 2008; Nicholson et al., 2012). Flagellin- and endotoxin-induced IBD accompanied by perturbation of the intestinal microbiome has been reported in human and rodent models (Imhann et al., 2017; Zhou et al., 2017; Schirmer et al., 2018; Zuo et al., 2018).

Feed composition is a major factor that influences intestinal bacterial composition in mammals (Muegge et al., 2011; Derrien and Veiga, 2017) and fishes (Ingerslev et al., 2014; Ni et al., 2014a; Miyake et al., 2015). Especially, significant changes in intestinal bacterial composition have been demonstrated in GCBB (Zhang et al., 2015). Therefore, we speculated that broad bean consumption alters the relative abundance of Gram-negative and flagellated bacteria in these fish, leading to induction of an epithelial inflammatory response and epithelial cell damage. To test this hypothesis, we compared the intestinal microbiota compositions of GCBB, grass carp fed hybrid giant napier (*Pennisetum sinese* Roxb, GCHG), and grass carp fed formula feed (GCFF) by sequencing the 16S rRNA gene. We also carried out a histological analysis of the intestinal inner wall by scanning electron microscopy, measured intestinal wall and serum TNF-α concentrations, and examined differences in relative abundance of genes encoding flagellin and endotoxin in intestinal microbiota of grass carp fed different diets.

## Materials and Methods

### Experimental Design and Protocol

This experiment was carried out at the Aquaculture Base of Pearl River Fisheries Research Institute over a period of 60 days. Grass carps weighing approximately 500 g from the same parents were collected from an artificial pond at Foshan Tongwei Fisheries Co. (Guangdong, China). After 2 months of being fed hybrid giant napier, formula feed, or marinated broad bean at 09:00 and 16:00 each day in the artificial pond, three carps in each diet group were collected and euthanized by submersion in 60 mg/L tricaine methane sulfonate solution. The tricaine methane sulfonate solution was prepared through dissolved tricaine methane sulfonate into equivalent sodium bicarbonate and then diluted to 60 mg/L using water from the culture tank. A 5 mL volume of blood was collected from the caudal vein of each fish with 10 mL asepsis injectors to measure serum TNF-α concentration. The fish were dissected on a sterile bench. A 0.5-cm piece of intestinal wall tissue and contents of the foregut (FG), midgut (MG), and hindgut (HG) of each fish (Ni et al., 2014b) were collected into a sterile 2 mL centrifuge tube and immediately frozen in liquid nitrogen until analysis of microbiota composition. The samples were designated based on the feed type and intestinal position—e.g., FFMG = microbiota collected from the MG of grass carp given formula feed. HG wall tissue samples were also collected in duplicate for measurement of TNF-α concentration in homogenates and for histological analysis of the intestinal inner wall by scanning electron microscopy.

The experiments protocols were approved by the Animal Ethics Committee of the Guangdong Provincial Zoological Society, China under permit number GSZ-AW001.

### Measurement of TNF-α Concentrations in Serum and Intestinal Wall

Blood samples were stored for 6 h at 4°C, then centrifuged for 20 min at 3,000 rpm/min and 4°C. The supernatant was collected and stored at -80°C until TNF-α concentration was measured.

Fat on the outer HG wall was removed and the tissue was cut open on a sterile bench and the contents removed. The tissue was washed for 30 s in phosphate-buffered saline (PBS) at 4°C to remove mucus, then cut into pieces and weighed. The pieces were transferred to a Tissuelyser-24, and nine times the volume of PBS buffer was added before homogenization for 30 s at 4°C. The homogenate was centrifuged for 20 min at 3,000 rpm and 4°C. The supernatant was collected and stored at -80°C until measurement of TNF-α concentration.

A TNF-α enzyme-linked immunosorbent assay (ELISA) kit (Cusabio Biotech, Wuhan, China) was used to measure TNF-α concentration according to the manufacturer’s instructions. Absorbance was read on a full-wavelength Multiskan GO microplate reader (Thermo Fisher Scientific, Waltham, MA, United States) with a sensitivity <50 pg/mL.

### Histological Analysis of Intestinal Inner Wall Tissue

The HG wall was cut open to reveal the inner wall, which was washed in 1% S-carboxymethyl-L-cysteine for 30 s to remove mucus. The intestinal wall was cut into 0.5 × 0.5-cm squares that were processed as previously described (Dimitroglou et al., 2009). After rinsing in phosphate buffer (pH 7.2), the squares were fixed in 2.5% glutaraldehyde for 18 h at 4°C. The samples were dehydrated in a graded series of ethanol as follows: 50% ethanol for 30 min, 70% ethanol for 45 min, 90% ethanol for 1 h, and 100% ethanol for 1 h, then subjected to three consecutive changes of ethanol and amyl acetate solution at 3:1, 2:2, and 1:3 ratios for 30 min each. The samples were left overnight in pure amyl acetate, then critical point dried in liquid nitrogen under vacuum. After coating with gold in an IB ion coater (Matsubo, Tokyo, Japan), the samples were visualized and photographed with an S-3400N scanning electron microscope (JEOL, Tokyo, Japan).

### Intestinal Microbiota DNA Extraction, Amplification, and Sequencing

Approximately 200 mg of intestinal content sample was homogenized with a 3 min bead-beating procedure at 30 Hz. Bacterial genomic DNA was extracted using an intestinal microbial DNA extraction kit (Omega Bio-tek, Norcross, GA, United States) according to the manufacturer’s recommendation. The quality and integrity of each DNA sample was determined by electrophoresis on a 1% agarose gel with 1× Tris-acetate-EDTA buffer. DNA concentration was quantified using a NanoDrop ND-2000 spectrophotometer (Thermo Fisher Scientific, Waltham, MA, United States).

PCR amplification of the V4 hypervariable region of the 16S rRNA gene was carried out using 515F and 806 R primers according to a previously reported method (Yan et al., 2016). The 30-μL reaction volume contained 15 μL of Phusion High-Fidelity PCR Master Mix (New England Biolabs, Ipswich, MA, United States), 0.2 μM forward and reverse primers, and about 10 ng of template DNA. The thermal cycling conditions were as follows: 98°C for 1 min; 30 cycles of 98°C for 10 s, 50°C for 30 s, and 72°C for 60 s; and 72°C for 5 min. PCR products were mixed with the same volume of 1× loading buffer (containing SYBR Green) and resolved by electrophoresis on a 2% agarose gel. Amplicons between 400 and 450 bp were selected for further experiments. The PCR products were mixed at an equidensity ratio and then purified using a GeneJET Gel Extraction kit (Thermo Fisher Scientific, Waltham, MA, United States).

Sequencing libraries were constructed using a Next Ultra DNA Library Prep kit (New England Biolabs) according to the manufacturer’s recommendations, and sample-specific barcodes were added. Library quality was assessed using a Qubit@ 2.0 Fluorometer (Thermo Fisher Scientific, Waltham, MA, United States) and Agilent Bioanalyzer 2100 system (Agilent Technologies, Santa Clara, CA, United States). The libraries were sequenced using the Illumina MiSeq platform, and 250 bp paired-end reads were generated. Sequencing was performed by Novogene Bioinformatics Technology (Beijing, China). Raw sequences were deposited in the National Center for Biotechnology Information Sequence Read Archive under accession number SRP080980.

### Sequencing Data Analysis

Sequences of the V4 region of the 16S rRNA gene were analyzed as previously described (Li et al., 2017; Ni et al., 2017). Briefly, paired-end reads were merged using FLASH software (Magoc and Salzberg, 2011). The merged tags were assigned to each sample according to the sample-specific barcodes. Chimera sequences were identified and removed using the Uchime algorithm before further analysis (Edgar et al., 2011). High-quality sequences were clustered into operational taxonomic units (OTUs) at 97% identity using UPARSE (Edgar, 2013; Xiang et al., 2018). Representative sequences for each OTU were selected and annotated with appropriate taxonomic information using the RDP Classifier tool (Wang et al., 2007). The open source software Phylogenetic Investigation of Communities by Reconstruction of Unobserved States (PICRUSt, v.1.0.0) was used to predict metagenome function based on 16S rRNA gene sequences (Langille et al., 2013). For the PICRUSt analysis, closed-reference OTUs were selected at 97% similarity against the Greengenes database^1^. The OTUs were normalized to predicted 16S rRNA copy number using PICRUSt with default parameters before gene family abundance was predicted for each metagenome based on Kyoto Encyclopedia of Genes and Genomes orthologous groups (Langille et al., 2013). The PICRUSt functional prediction was performed using Galaxy^2^. The relative abundances of genes involved in flagellar assembly were used to infer the changes of flagellated bacteria’s relative abundances.

### Statistical and Bioinformatics Analysis

Levene’s test was used to assess the homogeneity of variance of TNF-α concentrations. Potentially significantly different OTUs and predicted genes were identified using One-Way ANOVA within STAMP software (Parks et al., 2014). Then non-parametric Kruskal-Wallis rank sum test and pairwise comparisons were used to identify significantly different OTUs and predicted genes from the potentially significantly different OTUs and predicted genes using R 3.0.1 with base package^3^. The results are displayed as a heat map generated using caTools and gplots packages in R 3.0.1. Rarefaction curves were calculated for each sample with the QIIME software package (Caporaso et al., 2010), and drawn using an in-house Perl script. Taxonomy bar charts and the box-plots for the flagellum assembly genes were drew using R 3.0.1.

## Results

### Broad Bean Consumption Causes Epithelial Cell Damage and Intestinal Inflammation

**FIGURE 1:**
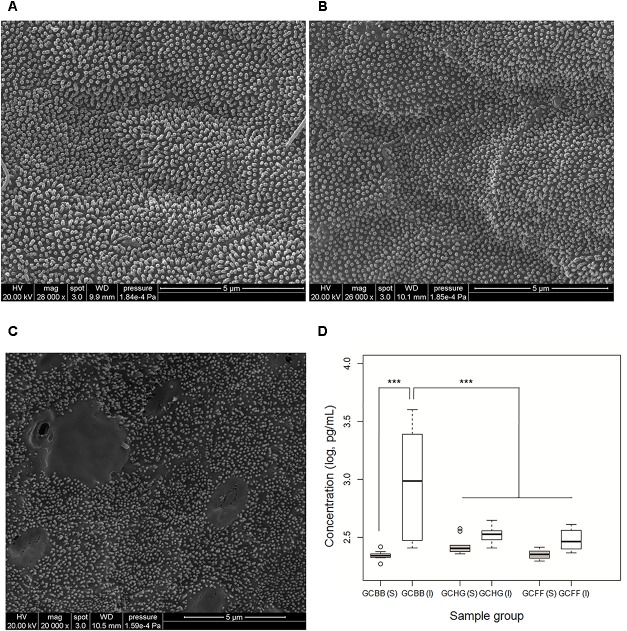
Epithelial cell damage and intestinal inflammation in grass carp fed different diets. **(A–C)** Epithelial cells of grass carp fed hybrid giant napier, formula feed, and broad bean, respectively, as observed by scanning electron microscopy. **(D)** TNF-α concentrations in serum [GCHG(S), GCFF(S), and GCBB(S)] and in intestinal wall [GCHG(I), GCFF(I), and GCBB(I)] of grass carp fed different diets. GCHG, GCFF, and GCBB indicate grass carp fed grass carp fed hybrid giant napier, formula feed, and broad bean, respectively. ^∗∗∗^*p* < 0.001.

Mucous epithelial cells in the GCHG and GCFF groups were covered by homogeneous brush border microvilli. However, the microvilli were irregular and discrete in GCBB tissue samples (**Figures 1A–C**). ELISA analysis showed that TNF-α concentration in the intestinal wall was higher in the GCBB than in the GCHG and GCFF groups, although no differences were observed in serum TNF-α levels (**Figure 1D**). These results indicate that a broad bean diet causes intestinal inflammation in grass carp.

### Intestinal Microbiota Composition Is Altered by Diet in Grass Carp

Totals of 1,324,523 (29,918–61,509) high-quality spliced sequences from 27 samples were obtained. Each sample was resampled for 29,918 bacterial sequences; 995 OTUs were detected according to the criterion of 97% sequence similarity. An average of 238 (93–601) OTUs were detected in each sample (**Supplementary Table S1**). Rarefaction curves (**Supplementary Figure S1**) showed that the sequence dataset represented the microbiome diversity and structure.

**FIGURE 2:**
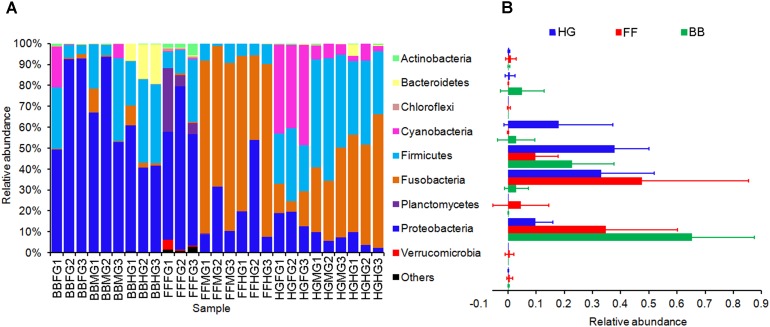
Phylum compositions of the gut microbiota in the grass carps fed with different feeds. In **(A)**, FFFG, FFMG, and FFHG indicate FG, MG, and HG microbiota, respectively, of grass carp fed formula feed; HGFG, HGMG, and HGHG indicate FG, MG, and HG microbiota, respectively, of grass carp fed hybrid giant napier; and BBFG, BBMG, and BBHG indicate FG, MG, and HG microbiota, respectively, of grass carp fed broad bean. In **(B)**, FF, HG, and BB indicate the gut microbiota from the grass carps fed with formula feed, hybrid giant napier, and broad bean, respectively.

There were 21 phyla represented in the intestinal microbiome of grass carps within the Archaea (*n* = 1) and Bacteria (*n* = 20) Domains, including Proteobacteria (36.65%), Fusobacteria (27.91%), Firmicutes (23.49%), Cyanobacteria (7.02%), Bacteroidetes (2.00%), and Planctomycetes (1.65%) were the predominant phyla (**Figure 2**), consistent with what has been reported in other fish species (Wu et al., 2012; Llewellyn et al., 2014; Ni et al., 2014a; Ghanbari et al., 2015).

**FIGURE 3:**
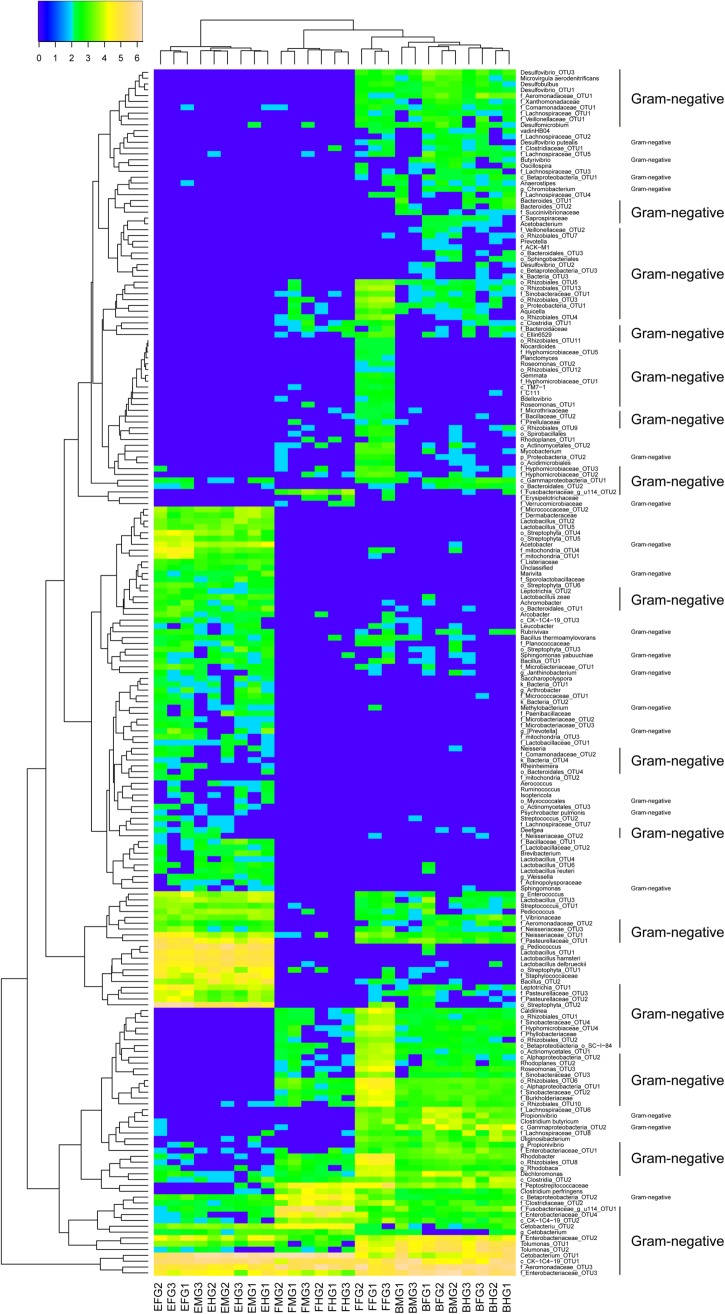
Heatmap of the relative abundances of all significantly different intestinal microbial OTUs in grass carp fed different diets (205 OTUs). The significant differences of the OTUs were tested using Kruskal-Wallis test by R software with the base package. Data were transformed according to the formula log_10_ (relative abundance × 100 + 1). FFFG, FFMG, and FFHG indicate FG, MG, and HG microbiota, respectively, of grass carp fed formula feed; HGFG, HGMG, and HGHG indicate FG, MG, and HG microbiota, respectively, of grass carp fed hybrid giant napier; and BBFG, BBMG, and BBHG indicate FG, MG, and HG microbiota, respectively, of grass carp fed broad bean.

A total of 205 OTUs (110 Gram-negative, 66 Gram-positive, and 31 unknown bacteria) differed significantly among intestinal microbiota of grass carp fed different diets (**Figure 3**). A total of 53 OTUs were increased in the GCBB group as compared to the other groups; most of these (34/53) were Gram-negative such as *Desulfovibrio* sp. and *Rhodobacter* sp. (**Figure 3**). On the other hand, most Gram-positive bacteria such as *Enterococcus* sp., *Bacillus* sp., and *Leucobacter* sp. were significantly reduced in the GCBB group (**Figure 3**). Compared to the GCHG group, the relative abundance of lactic acid bacteria (LAB) in the intestinal microbiome of the GCBB group was significantly decreased, although this was also true for the GCFF group (**Figure 3**).

### Relative Abundances of Genes Encoding Endotoxin and Flagellin Are Altered in Intestinal Microbiota of Grass Carp Fed Different Diets

**FIGURE 4:**
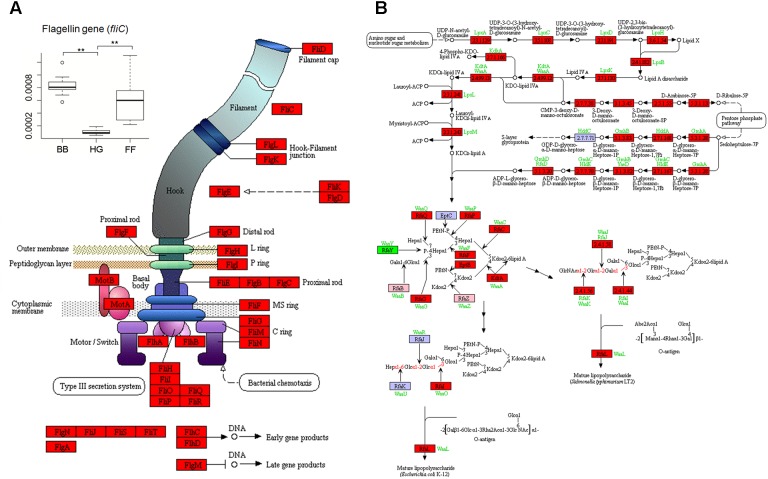
Kyoto Encyclopedia of Genes and Genomes pathways associated with genes related to flagellar assembly **(A)** and lipopolysaccharide biosynthesis **(B)** showing altered relative abundances in intestinal microbiota. The baseline data were showed in **Supplementary Table S2**. Red, green, and pink panels indicate genes showing significant increases and decreases and no significant difference in expression, respectively, in intestinal microbiota of grass carp fed broad bean. HG, FF, and BB indicate the gut microbiota of grass carp fed hybrid giant napier, formula feed, and broad bean, respectively.

According to the relative abundances of genes derived from predictions by PICRUSt, those genes involved in flagellar assembly were enriched in the GCBB group as compared to the other groups (**Figure 4** and **Supplementary Figure S2**). Relative abundance of the gene encoding flagellin (*fliC*)—an antigen of flagellated bacteria that can activate a mucosal inflammatory response (Gewirtz et al., 2001a,b; Zeng et al., 2003)—was higher in the GCBB than in the GCHG group (Levene test with multiple testing, *P* = 3.1 × 10^-6^; **Figure 3A**). Most (25/33) genes associated with the lipopolysaccharide (i.e., endotoxin) biosynthesis pathway were also enriched in the GCBB as compared to the other groups (*P* < 0.05; **Figure 4B** and **Supplementary Table S2**). The same trend was observed for five other genes, although the differences among groups were non-significant (**Figure 4B** and **Supplementary Table S2**).

## Discussion

Ingestion of chemicals or specific types of feed can induce intestinal inflammation in fish. Many studies have examined immune responses during intestinal inflammation (Urán et al., 2008; Chikwati et al., 2013; Hedrera et al., 2013). Intestinal inflammatory diseases in mammals are associated with changes in the composition of the intestinal microbiome (Gajardo et al., 2016; Marchesi et al., 2016; Chassaing et al., 2017), although there is little information on whether this is also true in fish.

A healthy mammalian cecum is characterized by the predominance of the phyla Firmicutes and Bacteroidetes, whereas an increase in the abundance of Gram-negative Enterobacteriaceae of Proteobacteria is a common marker of intestinal dysbiosis associated with intestinal inflammatory disease (Byndloss et al., 2017). In the present study, we observed that intestinal microbiota composition was altered in the GCBB group as compared to the GCHG and GCFF groups at phylum (**Figure 2**) and OTU (**Figure 3**) levels, which was accompanied by intestinal inflammation. Moreover, the abundances of Gram-negative and flagellated bacteria were higher whereas that of Gram-positive bacteria such as *Enterococcus* sp., *Bacillus* sp., and *Leucobacter* sp. was reduced in the GCBB group as compared to the other two groups (**Figure 3**). The latter species have been reportedly used as probiotics (Schrezenmeir and de Vrese, 2001; Ouwehand et al., 2002) that can suppress intestinal inflammation (Hughes et al., 2017). Additionally, the relative abundance of LAB was lower in the GCBB as compared to the GCHG group. LAB (e.g., *Lactobacillus plantarum* and *L. brevis*) regulate intestinal inflammation by inhibiting the phosphorylation of inhibitor of κB along with the transcription of interleukin-1β, TNF-α, and interferon-γ (Lee et al., 2008), and protect intestinal mucosa from injury by modulating TLR-2 signaling (Karczewski et al., 2010). Considering that increased ratio of Firmicutes and Bacteroidetes are associated with the obesity phenotype in human and rodent models (Ley et al., 2005; Moschen et al., 2012), the altered microbiota composition in phylum level in the present study showed that a ecological coherence of high bacterial taxonomic ranks (Philippot et al., 2010) probably existed in fish gut microbiota.

In human and mammal models, flagellated bacteria invade epithelial cells and induce intestinal inflammation (Ley and Gewirtz, 2016) by producing flagellin and endotoxin, which activate the expression of pro-inflammatory genes. The flagellated bacterium *Laribacter hongkongensis* isolated from patients with cirrhosis, bacteremia, and empyema plays a critical role in host intestinal inflammation (Yuen et al., 2001). Flagellated bacteria have also been linked to Crohn’s disease (Duck et al., 2007). TNF-α is a marker of inflammation; in damaged epithelial cells of grass carp, TNF-α level was upregulated 1 day after induction with trinitrobenzene sulfonic acid (Zhao et al., 2014). A similar phenomenon was reported in grass carp with *Aeromonas hydrophila*-induced intestinal inflammation (Song et al., 2014). It was suggested that broad bean consumption injures the intestinal mucosa of grass carp (Zhang et al., 2015). Consistent with these findings, our results showed that a diet consisting of broad bean significantly increased TNF-α concentration in the intestinal wall, leading to intestinal inflammation and epithelial cell damage.

Broad bean contains higher contents of amino acids and vitamins than wheat and corn (Fang et al., 1994; Wu and Bi, 2010). However, it also contains condensed tannins, polyphenols, convicine, soybean agglutinin (SBA), protease inhibitors and amylase inhibitors, which are anti-nutritional factors (Tacon, 1993). These anti-nutritional factors can significantly reduce growth and health of grass carp (Li et al., 2008). Even though there is no direct evidence to prove these anti-nutritional factors can change the gut microbiota of grass carp, SBA is likely to change gut microbial composition (Feng and Zhou, 2006; Wu et al., 2007). However, the internal mechanism of SBA changing gut microbial composition should be further studied.

In summary, we demonstrated that consumption of broad bean altered the intestinal microbiota composition of grass carp, increasing the relative abundances of Gram-negative and flagellated bacteria. These changes resulted in epithelial cell damage and intestinal inflammation in the GCBB group. Our results suggest that broad bean-induced intestinal inflammation in grass carp can be used as a model to investigate the mechanism of IBD.

## Author Contributions

ZL, JX, and EY designed the experiments. ZL, GW, and WG performed the experiments. ZL, EY, and KZ analyzed the data. ZL, EY, and DY wrote the paper.

## Conflict of Interest Statement

The authors declare that the research was conducted in the absence of any commercial or financial relationships that could be construed as a potential conflict of interest.

## Footnotes

1 http://greengenes.secondgenome.com/downloads/database/13_5

2 http://huttenhower.sph.harvard.edu/galaxy/

3 https://www.r-project.org/
