# Supplementary material for: Broad Bean (Vicia faba L.) Induces Intestinal Inflammation in Grass Carp (Ctenopharyngodon idellus C. et V) by Increasing Relative Abundances of Intestinal Gram-Negative and Flagellated Bacteria
Source: Front Microbiol. 2018 Aug 17;9:1913. doi: 10.3389/fmicb.2018.01913 (PMC6107684; doi:10.3389/fmicb.2018.01913)

Broad Bean (*Vicia faba* L.) Initiates Intestinal Inflammation in Grass Carp (*Ctenopharyngodon idellus* C. et V) by Increasing Relative Abundances of Intestinal Gram-negative and Flagellated Bacteria

Zhifei Li[1,2], Ermeng Yu[1,2*], Guangjun Wang[1,2], Deguang Yu[1,2], Kai Zhang[1,2], Wangbao Gong[1,2], Jun Xie[1,2*]

***Correspondence:** Dr. Jun Xie (xiejunhy01@126.com) and Dr. Ermeng Yu (yem@prfri.ac.cn)

[1]Key Laboratory of Tropical and Subtropical Fishery Resource Application and Cultivation, Pearl River Fisheries Research Institute, Chinese Academy of Fishery Science, Guangzhou, China,

[2]Guangdong Ecological Remediation of Aquaculture Pollution Research Center, Guangzhou, China

Table S1 Sequencing results for gut microbiota in grass carps fed with different feeds.

Sample name	Raw sequence	Clean sequence	Effective sequence	Average Length (nt)	Q30	OUT	Chao1 index	Shannon index
BBFG1	53,694	53,648	52,080	253	97.02	369	482.438	3.889
BBFG2	62,861	62,813	59,808	253	97.06	303	402.02	2.752
BBFG3	63,862	63,821	61,509	253	97.04	258	333.146	2.914
BBHG1	55,159	55,115	52,782	253	97.13	277	418.973	3.533
BBHG2	54,108	54,075	50,859	253	97.13	291	350.381	3.57
BBHG3	63,421	63,375	60,402	253	97	310	415	3.208
BBMG1	32,765	32,751	31,456	253	96.83	196	222.053	2.587
BBMG2	55,120	55,080	53,300	253	96.96	376	487.342	2.598
BBMG3	61,433	61,378	58,181	253	97.09	249	352.837	2.606
HGFG1	60,563	59,474	57,180	253	96.81	193	226	3.424
HGFG2	59,216	58,123	55,135	253	96.85	169	195.464	3.604
HGFG3	60,928	58,339	55,784	253	96.57	156	201.042	3.055
HGHG1	62,086	60,662	57,635	253	96.98	209	246.561	3.366
HGHG2	60,888	58,019	55,007	253	96.71	126	165	2.704
HGHG3	59,094	57,268	55,248	252	96.88	198	265.097	2.408
HGMG1	60,470	59,068	55,414	253	96.83	154	211.5	3.292
HGMG2	63,166	60,581	57,823	253	96.8	135	201	3.147
HGMG3	59,786	57,759	54,831	253	96.76	129	149.308	2.898
FFFG1	47,082	47,034	44,903	253	96.79	601	646.267	6.45
FFFG2	35,131	35,099	31,037	253	96.84	492	525.632	4.547
FFFG3	54,929	54,884	44,153	253	97.15	577	626.189	6.029
FFHG1	34,651	34,636	32,466	252	97.01	99	118.333	1.873
FFHG2	43,641	43,617	39,371	253	97.04	93	118.87	2.071
FFHG3	51,364	51,322	47,390	252	97.12	103	146.938	2.025
FFMG1	45,386	45,357	35,722	252	97.08	156	188.621	1.686
FFMG2	31,638	31,624	30,026	252	97.13	119	158.2	1.697
FFMG3	39,038	39,008	35,021	252	97.07	100	113.5	2.001

FFFG, FFMG, and FFHG indicate FG, MG, and HG microbiota, respectively, of grass carp fed formula feed; HGFG, HGMG, and HGHG indicate FG, MG, and HG microbiota, respectively, of grass carp fed hybrid giant napier; and BBFG, BBMG, and BBHG indicate FG, MG, and HG microbiota, respectively, of grass carp fed broad bean.

Table S2 Statistical significance level of genes involved in the lipopolysaccharide biosynthesis among different grass carp groups. HG, FF, and BB indicate the gut microbiota of grass carp fed hybrid giant napier, formula feed, and broad bean, respectively. Relative abundances of the genes were derived from PICRUSt predictions based on bacterial 16S rRNA gene reads.

Gene ID	Mean ± S.E.			Kruskal-Wallis rank sum test		*P* value		
	BB	HG	FF	Kruskal-Wallis Chi-squared	*P*-value	BB vs. HG	BB vs. FF	HG vs. FF
K02407	$3.53×10^{-4}±3.43×10^{-5}$	$4.41×10^{-5}±2.24×10^{-5}$	$1.66×10^{-4}±1.03×10^{-4}$	21.2099	$2.48×10^{-5}$	$1.1×10^{-9}$	$7.4×10^{-6}$	$1.4×10^{-3}$
K02406	$8.41×10^{-4}±1.76×10^{-4}$	$1.03×10^{-4}±4.16×10^{-5}$	$6.27×10^{-4}±3.76×10^{-4}$	18.5644	$9.31×10^{-5}$	$3.1×10^{-6}$	0.213	$3.4×10^{-4}$
K02397	$3.27×10^{-4}±4.03×10^{-5}$	$4.10×10^{-5}±2.04×10^{-5}$	$1.97×10^{-4}±1.38×10^{-4}$	16.8924	$2.15×10^{-4}$	$5.4×10^{-7}$	$9.6×10^{-3}$	$1.8×10^{-3}$
K02396	$3.75×10^{-4}±7.20×10^{-5}$	$4.45×10^{-5}±2.49×10^{-5}$	$2.22×10^{-4}±1.68×10^{-4}$	16.5538	$2.54×10^{-4}$	$2.4×10^{-6}$	0.0165	$4.9×10^{-3}$
K02414	$3.06×10^{-4}±6.63×10^{-5}$	$3.04×10^{-5}±1.28×10^{-5}$	$1.32×10^{-4}±8.00×10^{-5}$	20.6667	$3.25×10^{-5}$	$2.9×10^{-9}$	$8.0×10^{-6}$	$4.7×10^{-3}$
K02389	$3.67×10^{-4}±6.52×10^{-5}$	$4.35×10^{-5}±2.40×10^{-5}$	$2.32×10^{-4}±1.65×10^{-4}$	17.2381	$1.81×10^{-4}$	$2.1×10^{-6}$	0.0316	$2.2×10^{-3}$
K02390	$3.85×10^{-4}±6.42×10^{-5}$	$4.46×10^{-5}±2.52×10^{-5}$	$2.22×10^{-4}±1.69×10^{-4}$	16.5538	$2.54×10^{-4}$	$1.4×10^{-6}$	$9.8×10^{-3}$	$4.6×10^{-3}$
K02392	$4.21×10^{-4}±1.14×10^{-4}$	$5.37×10^{-5}±3.51×10^{-5}$	$2.69×10^{-4}±2.03×10^{-4}$	17.0265	$2.01×10^{-4}$	$2.0×10^{-5}$	0.0781	$7.9×10^{-3}$
K02391	$3.15×10^{-4}±6.49×10^{-5}$	$3.32×10^{-5}±1.50×10^{-5}$	$1.85×10^{-4}±1.26×10^{-4}$	18.6667	$8.84×10^{-5}$	$5.1×10^{-7}$	0.008	0.002
K02557	$4.66×10^{-4}±9.14×10^{-5}$	$6.10×10^{-5}±3.05×10^{-5}$	$3.82×10^{-4}±2.57×10^{-4}$	16.8995	$2.14×10^{-4}$	$4.2×10^{-5}$	0.809	$7.4×10^{-4}$
K02556	$4.77×10^{-4}±9.68×10^{-5}$	$5.75×10^{-5}±2.81×10^{-5}$	$3.19×10^{-4}±1.85×10^{-4}$	18.2963	$1.06×10^{-4}$	$4.4×10^{-7}$	0.0333	$3.7×10^{-4}$
K02393	$3.22×10^{-4}±5.99×10^{-5}$	$3.33×10^{-5}±1.50×10^{-5}$	$2.01×10^{-4}±1.45×10^{-4}$	17.5062	$1.58×10^{-4}$	$1.7×10^{-6}$	0.027	0.002

K02394	$3.22\times10^{-4}\pm5.99\times10^{-5}$	$3.32\times10^{-5}\pm1.50\times10^{-5}$	$2.01\times10^{-4}\pm1.45\times10^{-4}$	17.5062	1.58×10^{-4}	1.7×10^{-6}	0.028	0.002
K02408	$3.85\times10^{-4}\pm6.42\times10^{-5}$	$4.43\times10^{-5}\pm2.53\times10^{-5}$	$2.31\times10^{-4}\pm1.64\times10^{-4}$	17.2381	1.81×10^{-4}	8.4×10^{-7}	0.012	0.0022
K02387	$3.88\times10^{-4}\pm6.33\times10^{-5}$	$4.47\times10^{-5}\pm2.51\times10^{-5}$	$2.36\times10^{-4}\pm1.69\times10^{-4}$	17.2381	1.81×10^{-4}	1.1×10^{-6}	0.016	0.0023
K02388	$3.86\times10^{-4}\pm6.43\times10^{-5}$	$4.44\times10^{-5}\pm2.53\times10^{-5}$	$2.36\times10^{-4}\pm1.69\times10^{-4}$	17.2381	1.81×10^{-4}	1.3×10^{-6}	0.0176	0.0024
K02409	$3.55\times10^{-4}\pm3.43\times10^{-5}$	$4.14\times10^{-5}\pm2.06\times10^{-5}$	$2.26\times10^{-4}\pm1.56\times10^{-4}$	16.8889	2.15×10^{-4}	6.7×10^{-7}	0.0216	9.6×10^{-4}
K02410	$3.85\times10^{-4}\pm6.42\times10^{-5}$	$4.43\times10^{-5}\pm2.53\times10^{-5}$	$2.38\times10^{-4}\pm1.72\times10^{-4}$	17.2381	1.81×10^{-4}	1.6×10^{-6}	0.0221	0.0024
K02416	$3.84\times10^{-4}\pm6.42\times10^{-5}$	$4.29\times10^{-5}\pm2.43\times10^{-5}$	$2.31\times10^{-4}\pm1.63\times10^{-4}$	17.2381	1.81×10^{-4}	7.6×10^{-7}	0.012	0.002
K02417	$3.60\times10^{-4}\pm3.39\times10^{-5}$	$4.30\times10^{-5}\pm2.34\times10^{-5}$	$2.39\times10^{-4}\pm1.63\times10^{-4}$	16.8889	2.15×10^{-4}	1.1×10^{-6}	0.043	7.9×10^{-4}
K02400	$3.84\times10^{-4}\pm6.39\times10^{-5}$	$4.40\times10^{-5}\pm2.53\times10^{-5}$	$2.33\times10^{-4}\pm1.66\times10^{-4}$	17.2381	1.81×10^{-4}	1.0×10^{-6}	0.015	0.0022
K02401	$3.80\times10^{-4}\pm6.37\times10^{-5}$	$4.31\times10^{-5}\pm2.51\times10^{-5}$	$2.22\times10^{-4}\pm1.71\times10^{-4}$	16.8924	2.15×10^{-4}	1.8×10^{-6}	0.013	0.0047
K13820	$4.20\times10^{-7}\pm4.08\times10^{-7}$	$1.11\times10^{-8}\pm2.42\times10^{-8}$	$5.10\times10^{-7}\pm7.19\times10^{-7}$	15.6992	3.90×10^{-4}	0.25	1.00	0.11
K02411	$3.48\times10^{-4}\pm3.44\times10^{-5}$	$3.93\times10^{-5}\pm1.98\times10^{-5}$	$2.00\times10^{-4}\pm1.28\times10^{-4}$	19.7072	5.25×10^{-5}	3.4×10^{-8}	0.0014	5.7×10^{-4}
K02412	$3.82\times10^{-4}\pm6.44\times10^{-5}$	$4.27\times10^{-5}\pm2.42\times10^{-5}$	$2.31\times10^{-4}\pm1.63\times10^{-4}$	17.2381	1.81×10^{-4}	8.4×10^{-7}	0.013	0.0021
K02418	$3.47\times10^{-4}\pm6.95\times10^{-5}$	$4.02\times10^{-5}\pm2.24\times10^{-5}$	$1.46\times10^{-4}\pm9.10\times10^{-5}$	21.2099	2.48×10^{-5}	2.8×10^{-9}	4.4×10^{-6}	0.0084
K02419	$3.85\times10^{-4}\pm6.42\times10^{-5}$	$4.40\times10^{-5}\pm2.52\times10^{-5}$	$2.37\times10^{-4}\pm1.72\times10^{-4}$	17.2381	1.81×10^{-4}	1.6×10^{-6}	0.022	0.0023
K02420	$3.83\times10^{-4}\pm6.44\times10^{-5}$	$4.37\times10^{-5}\pm2.51\times10^{-5}$	$2.37\times10^{-4}\pm1.71\times10^{-4}$	17.2381	1.81×10^{-4}	1.7×10^{-6}	0.024	0.0023
K02421	$3.80\times10^{-4}\pm6.37\times10^{-5}$	$4.31\times10^{-5}\pm2.51\times10^{-5}$	$2.22\times10^{-4}\pm1.71\times10^{-4}$	16.5538	2.54×10^{-4}	1.8×10^{-6}	0.013	0.0047

K02399	$3.03\times10^{-4}\pm6.77\times10^{-5}$	$2.96\times10^{-5}\pm1.41\times10^{-5}$	$1.15\times10^{-4}\pm7.93\times10^{-5}$	20.6455	3.29×10^{-5}	3.7×10^{-9}	2.7×10^{-6}	0.019
K02386	$3.10\times10^{-4}\pm6.44\times10^{-5}$	$3.18\times10^{-5}\pm1.44\times10^{-5}$	$1.83\times10^{-4}\pm1.24\times10^{-4}$	18.2998	1.06×10^{-4}	4.8×10^{-7}	0.0086	0.0017
K02413	$3.49\times10^{-4}\pm7.01\times10^{-5}$	$4.10\times10^{-5}\pm2.39\times10^{-5}$	$1.63\times10^{-4}\pm0.89\times10^{-4}$	21.7390	1.90×10^{-5}	2.3×10^{-9}	1.3×10^{-5}	0.0022
K02422	$3.81\times10^{-4}\pm6.53\times10^{-5}$	$4.37\times10^{-5}\pm2.61\times10^{-5}$	$1.85\times10^{-4}\pm1.00\times10^{-4}$	21.7390	1.90×10^{-5}	1.2×10^{-9}	1.4×10^{-5}	8.8×10^{-4}
K02423	$2.16\times10^{-4}\pm7.68\times10^{-5}$	$2.46\times10^{-5}\pm1.50\times10^{-5}$	$4.40\times10^{-5}\pm3.90\times10^{-5}$	17.5062	1.58×10^{-4}	8.4×10^{-8}	5.2×10^{-7}	1.00
K02402	$2.31\times10^{-4}\pm7.45\times10^{-5}$	$1.92\times10^{-5}\pm1.14\times10^{-5}$	$4.17\times10^{-5}\pm3.68\times10^{-5}$	17.7460	1.40×10^{-4}	6.3×10^{-9}	5.0×10^{-8}	1.00
K02403	$2.13\times10^{-4}\pm7.67\times10^{-5}$	$1.94\times10^{-5}\pm1.15\times10^{-5}$	$4.07\times10^{-5}\pm3.54\times10^{-5}$	17.7460	1.40×10^{-4}	4.3×10^{-8}	3.3×10^{-7}	1.00
K02398	$3.07\times10^{-4}\pm6.61\times10^{-5}$	$3.66\times10^{-5}\pm1.64\times10^{-5}$	$1.46\times10^{-4}\pm8.66\times10^{-5}$	20.1305	4.25×10^{-5}	1.1×10^{-8}	4.8×10^{-5}	0.004

Fig. S1 Rarefaction curves of different sample OTUs. FFFG, FFMG, and FFHG indicate FG, MG, and HG microbiota, respectively, of grass carp fed formula feed; HGFG, HGMG, and HGHG indicate FG, MG, and HG microbiota, respectively, of grass carp fed hybrid giant napier; and BBFG, BBMG, and BBHG indicate FG, MG, and HG microbiota, respectively, of grass carp fed broad bean.

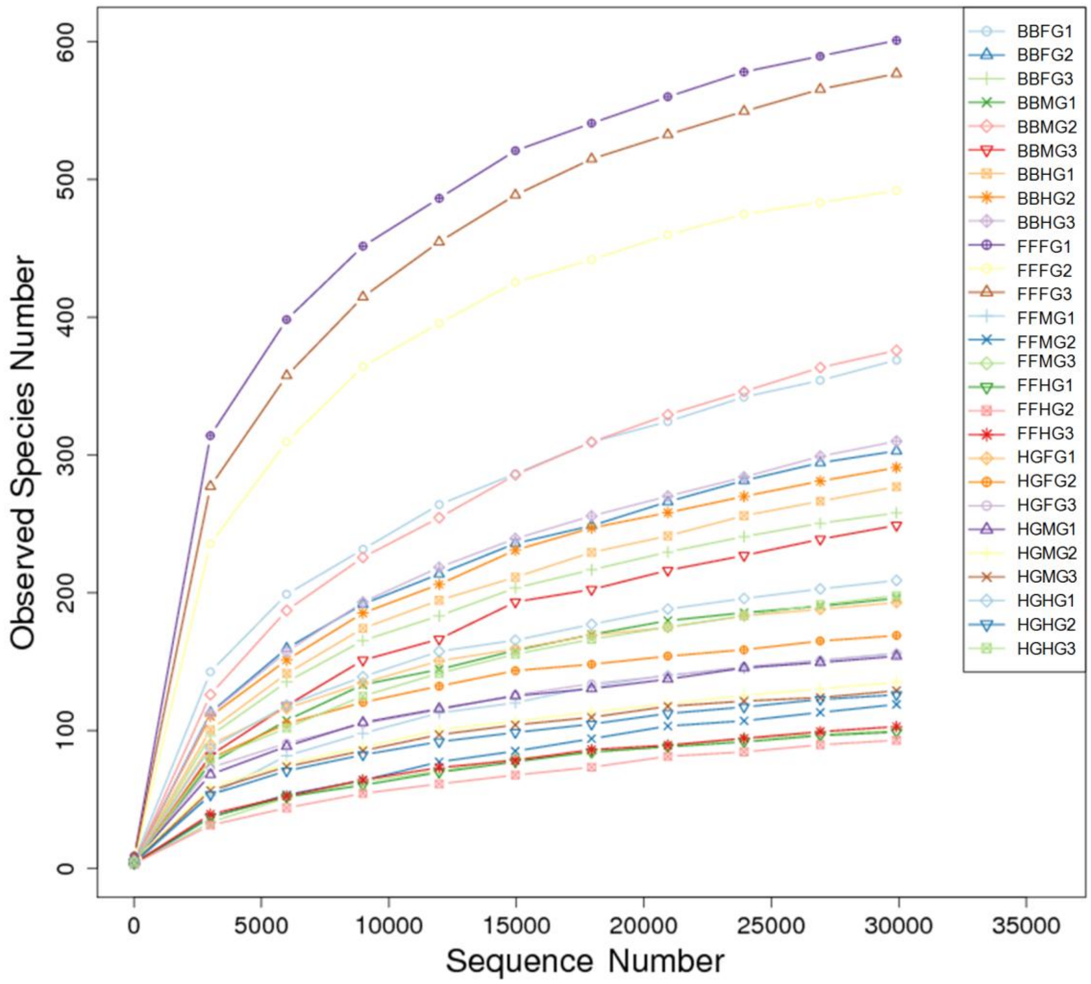

Fig. S2 Relative abundance of the genes involved in the flagellar assembly in the gut microbiota of grass carps fed with different feeds. FF, HG, and BB indicate the gut microbiota from the grass carps fed with formula feed, hybrid giant napier, and broad bean, respectively.

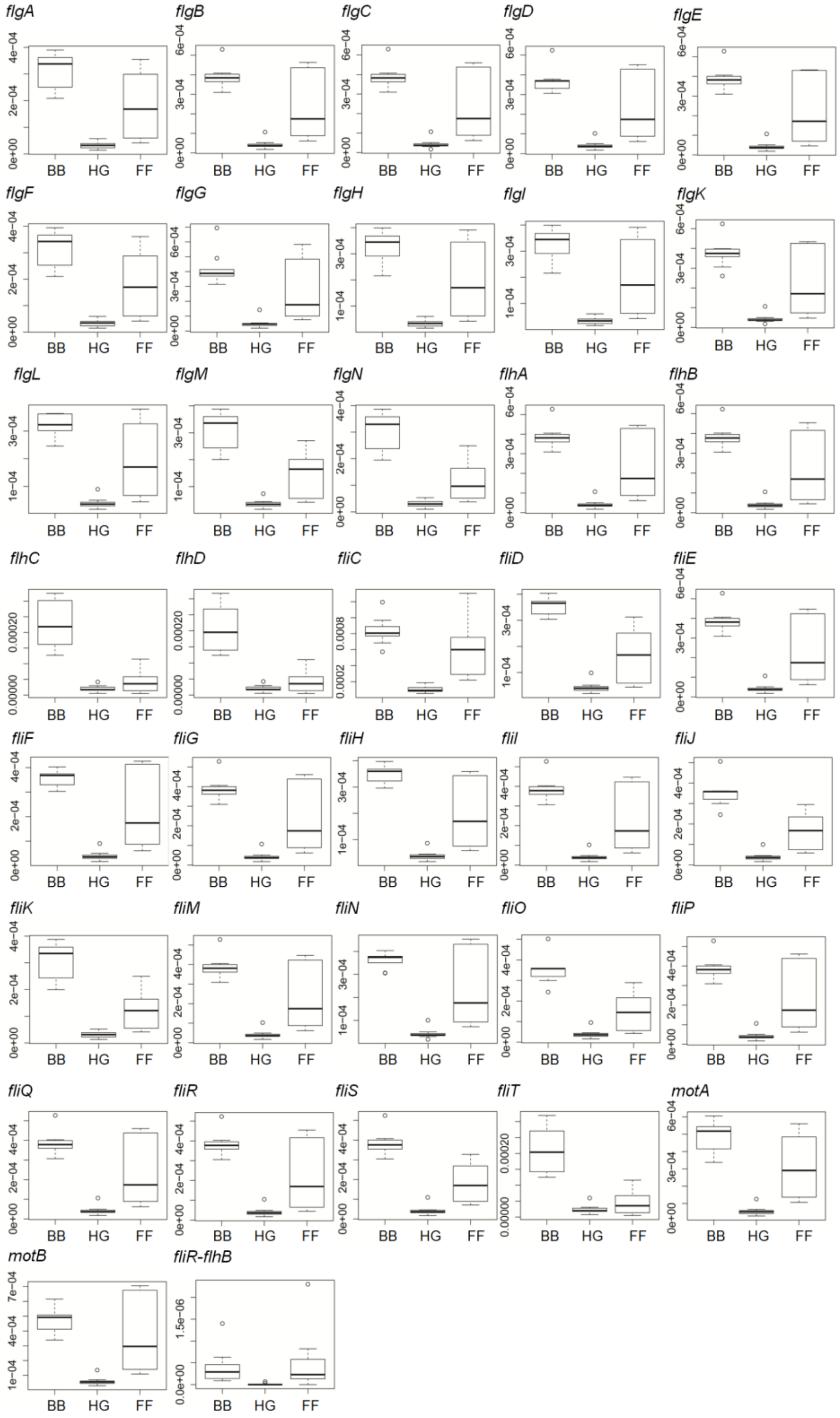

Supplement: Supplementary file 1 [file Data_Sheet_1.pdf]
